# Discovery of Dome‐Shaped Superconducting Phase and Anisotropic Transport in a van der Waals Layered Candidate NbIrTe_4_ under Pressure

**DOI:** 10.1002/advs.202103250

**Published:** 2021-11-01

**Authors:** Meiling Jin, Peng Yu, Changzeng Fan, Qiang Li, Panlong Kong, Zhiwei Shen, Xiaomei Qin, Zhenhua Chi, Changqing Jin, Guangtong Liu, Guyue Zhong, Gang Xu, Zheng Liu, Jinlong Zhu

**Affiliations:** ^1^ Department of Physics and Shenzhen Engineering Research Center for Frontier Materials Synthesis at High Pressures Southern University of Science and Technology (SUSTech) Shenzhen 518055 China; ^2^ Center for High Pressure Science and Technology Advanced Research (HPSTAR) Beijing 100094 China; ^3^ State Key Laboratory of Optoelectronic Materials and Technologies School of Materials Science and Engineering Sun Yat‐sen University Guangzhou 510275 China; ^4^ State Key Laboratory of Metastable Materials Science and Technology Yanshan University Qinhuangdao 066004 China; ^5^ Department of Physics Shanghai Normal University Shanghai 200234 China; ^6^ Institute of High Pressure Physics School of Physical Science and Technology Ningbo University Ningbo 315211 China; ^7^ Beijing National Laboratory for Condensed Matter Physics Institute of Physics Chinese Academy of Sciences School of Physical Sciences University of Chinese Academy of Sciences Beijing 100190 China; ^8^ Wuhan National High Magnetic Field Center and School of Physics Huazhong University of Science and Technology Wuhan 430074 China; ^9^ School of Materials Science and Engineering Nanyang Technological University 50 Nanyang Avenue Singapore 639798 Singapore; ^10^ School of Electrical and Electronic Engineering Nanyang Technological University Singapore 639798 Singapore

**Keywords:** anisotropic transport, high pressure, phase transition, superconductivity

## Abstract

The unique electronic structure and crystal structure driven by external pressure in transition metal tellurides (TMTs) can host unconventional quantum states. Here, the discovery of pressure‐induced phase transition at ≈2 GPa, and dome‐shaped superconducting phase emerged in van der Waals layered NbIrTe_4_ is reported. The highest critical temperature (*T*_c_) is ≈5.8 K at pressure of ≈16 GPa, where the interlayered Te–Te covalent bonds form simultaneously derived from the synchrotron diffraction data, indicating the hosting structure of superconducting evolved from low‐pressure two‐dimensional (2D) phase to three‐dimensional (3D) structure with pressure higher than 30 GPa. Strikingly, the authors have found an anisotropic transport in the vicinity of the superconducting state, suggesting the emergence of a “stripe”‐like phase. The dome‐shaped superconducting phase and anisotropic transport are possibly due to the spatial modulation of interlayer Josephson coupling .

## Graphical abstract

The correlation of novel electrical properties and structural evolutions in van der Waals layered material is revealed by performing special high‐pressure measurements. A superconducting model is proposed to explain the emergence of “stripe”‐like phases and the evolution of critical temperature. Spatial modulation of interlayer Josephson coupling furthers the understanding of the complex relationships between structure, anisotropy, and superconductivity.

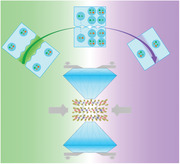

## 1 Introduction

Two‐dimensional (2D) or layered structures can hold rich quantum physics, such as 2D electron systems, Weyl semimetals, topological materials, superconductivity, and topological superconductivity, which can potential hold the Majorana fermion, and so on. Since 1990s, researchers already started to fabricate lower dimensional materials, such as monolayer cuprate superconductors,^[^ ^1^, ^2^ ^]^ deposition of metallic thin films of single elemental superconductors^[^ ^3^ ^]^ and more recently, highly‐crystalline^[^ ^4^ ^]^ atomic layers can be obtained by molecular beam epitaxy or through exfoliation of crystalline layered parent compounds. Then the free‐standing monolayer of Bi2212, Bi_2_Sr_2_CaCu_2_O_8_,^[^ ^5^ ^]^ was fabricated and reported to hold all the fundamental physics and to be indistinguishable from its parent bulk. To the contrary, the monolayered transition metal dichalcogenides (TMD) NbSe_2_ gives a much‐reduced critical temperature (*T*_c_) compared with its bulk crystal.^[^ ^6^ ^]^ On the other hand, the TMDTaS_2_^[^ ^7^ ^]^ in the thin film form hold an increased *T*_c_ of 2.2 K compared with that of 0.5 K in bulk. So the interactions between different van der Waals layers, and resulted property evolution as a function of film thickness, such as phase transition, enhanced/weaker Coulomb interaction, band structure change, and interlayer Cooper pair interactions, make 2D TMD systems electronic platform with fruitful physics. It is reminiscent of the coupling between a carrier storage layer and carrier conducting layer in the unconventional superconductivity. In most high‐*T*_c_ cuprates, the octahedron is elongated along the *c* axis, leading to a 3d*_x_*_2‐_ *_y_*_2_ orbital at the top of the band structure with 2D characteristic. A recent exception is Ba_2_CuO_4 −_ *_y_* with a compressed version and the 3d_3_ *_z_*_2‐_ *_r_*_2_ orbital lifted above the 3d*_x_*_2‐_ *_y_*_2_ orbital, resulting in a temperature of 30 K higher than the *T*_c_ for the isostructural counterparts, and a significant three‐dimensional 3D nature.^[^ ^8^ ^]^ So the relationship of crystal structure versus superconducting *T*_c_ is still needed to be further explored. Specifically, the interaction and proximity effect between TMD individual layers and between epitaxy layers and substrates bring more complex parameters in, therefore a symmetrical thickness dependent evolution and external field tuning of free‐standing layered materials can disclose more knowledge of fundamental mechanism and guide 2D device application.

High pressure is an important tool to regulate physical properties of condensed matter by directly changing lattice parameters and anisotropy without introducing impurities, therefore is still an effective reversible in situ approach to effectively tune interlayer coupling, electronic structure, order parameter, and dimensionality of TMD van der Waals layered structures. Successfully, high pressure has been applied to induce quantum phase transitions in topological materials, such as metallization and superconductivity in topological insulators Bi_2_Se_3_^[^ ^9^, ^10^ ^]^ and Bi_2_Te_3_,^[^ ^11^, ^12^ ^]^ and topological semimetals.^[^ ^13^, ^14^, ^15^, ^16^ ^]^ External high pressure has also been applied in Moiré superlattices by tuning the layer hybridization regardless of precise twist angle control.^[^ ^17^ ^]^ High pressure can also be directly applied to tune the interlayer coupling to demonstrate expected *B*_C_ oscillation in van der Waals TMD crystals,^[^ ^18^ ^]^ or squeeze gas molecular to be intercalated between layers to enhance the in‐plane *B*_C_.

Over recent years, transition metal chalcogenides (TMCs) have become one of the materials, which have been extensively investigated due to their unique properties, including tunable electronic transport properties, high mechanical flexibility, and their potential applications in fabricated devices. As important members of TMCs, transition metal tellurides (TMTs) have drawn much attention for their various crystal structures with rich physical properties. Pressure‐induced dome‐shaped superconducting phase was observed in type‐II Weyl semimetal candidates T*_d_*‐WTe_2_^[^ ^14^ ^]^ and T*_d_*‐MoTe_2_.^[^ ^16^ ^]^ The ternary TMTNbIrTe_4_, an ordered variant of the WTe_2_, is a predicted time‐reversal invariant type‐II Weyl semimetal candidate with ambient T*_d_*‐phase.^[^ ^19^ ^]^ The recent emerging layered topological materials and its related superconductivity have offered a unique platform for exploring related mechanism at the quantum critical boundaries. However, most studies of layered materials performed within the individual layer or parallel to the layered direction. Therefore, to explore the anisotropic properties, including a vertical direction to the layers is important to help explore the mechanism from multiple perspectives. Here we report pressure‐induced dome‐shaped superconducting in NbIrTe_4_ with an emerging *T*_c_ of ≈2.5 K at ≈2 GPa and a maximum *T*_c_ of ≈5.8 K at ≈16 GPa. We found anisotropic behavior just before entering the superconducting state demonstrated by transport measurements, which suggests a “stripe”‐like phase due to spatially modulated interlayer interaction. The enhanced coupling is called to be responsible for the global superconducting at ≈5 GPa, maximum *T*_c_ at ≈16 GPa; and pressure induced degree of disordering for the high pressure “stripe phase.” These anisotropic properties and their relationships with interlayer coupling provide a potential pathway of studying/identifying the minimized functional block holding various quantum states and, designing of 2D devices through the understanding of proximity effects, charge transfer, stresses, and even magnetic exchanges addressed by the bilateral nearest layers or substrates holding the 2D thin films.

## 2 Results and Discussion

**Figure 1:**
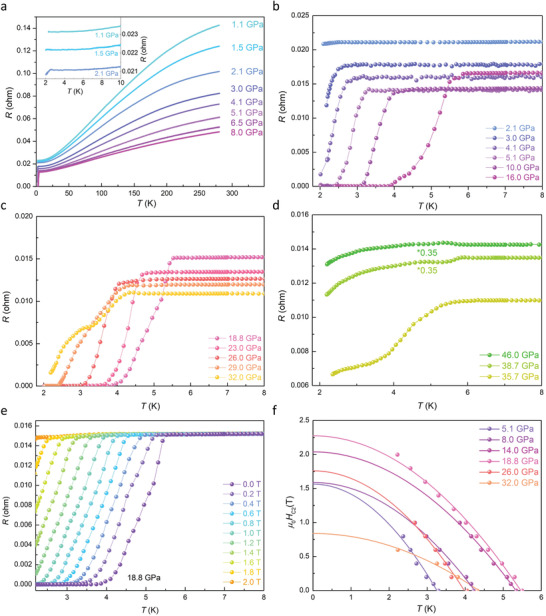
Electrical transport measurements of NbIrTe_4_ at high pressures. a) The plot of electrical resistance as a function of temperature measured without magnetic field for the pressures ranging from 1.1 to 8.0 GPa. The inset shows detail of data below 10 K with a hint of superconductivity at 2.1 GPa. b–d) Temperature dependence of electrical resistance measured at different pressures range from 2.1 to 16 GPa, 18.8 to 32.0 GPa, and 35.7 to 46.0 GPa, respectively. e) Temperature dependence of electrical resistance at fixed pressures under different magnetic fields perpendicular to *ab*‐plane. f) The fitted *µ*_0_ *H*_c2_ perpendicular to *ab*‐plane at different pressures. The solid lines represent the WHH fits.

NbIrTe_4_ is a layered TMTs material with an orthorhombic symmetry (space group of *Pmn*2_1_^[^ ^20^ ^]^), and exhibits similar atomic structure to T*_d_*‐WTe_2_,^[^ ^14^ ^]^ T*_d_*‐MoTe_2_^[^ ^16^ ^]^ reported previously. At ambient pressure, a metal‐like behavior indicated by the temperature‐dependence of resistivity was dominated in the Weyl‐semimetal state and no hint of any superconductivity was observed down to 1.6 K, as shown in Figure S1, Supporting Information. By applying high pressure, the temperature dependence of electrical resistance in *ab*‐plane is shown in **Figure** **1**a–d. Intriguingly, at pressure of 2.1 GPa, the resistance drops abruptly at 2.5 K but not to zero resistance by further decreasing temperature, indicating an emergence of new quantum state, like strange metal or granular superconductivity. The drop in resistance becomes more pronounced at higher pressure, and eventually zero resistance is achieved at pressures higher than 5.1 GPa (see Figure 1b), confirming the superconductivity. The *T*_c_ culminates 5.8 K at ≈16 GPa followed by a monotonically decrease down to 4.2 K at 32.0 GPa (see Figure 1c). Upon further compression, *T*_c_ regains an increment to ≈5.8 K at 38.7 GPa (see Figure 1d). To substantiate the superconducting nature, magnetic field dependence of resistance as a function of temperature is measured (see Figure 1e). The zero‐temperature upper critical field can be estimated to be 1.56 T at 5.1 GPa, 2.27 T at 18.8 GPa, and 0.83 T at 32.0 GPa, respectively, by fitting the data with Werthamer–Helfand–Hohenberg (WHH) formula^[^ ^21^ ^]^ (see Figure 1f). When pressure is higher than 32.0 GPa, there is a two‐step drop of resistance with no zero resistance, and the upper critical field as a function of temperature at that pressure showed a different trend.

**Figure 2:**
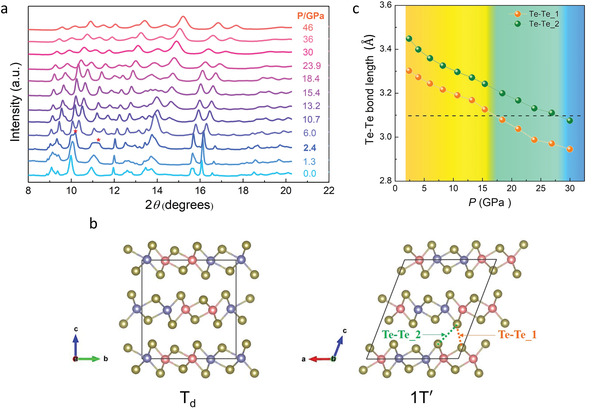
Synchrotron X‐ray diffraction data for NbIrTe_4_ powders and changes of lattice parameters at different pressures. a) The evolution of diffraction patterns over sample compression at room temperature. b) T*_d_*‐NbIrTe_4_ and 1 T′‐NbIrTe_4_ crystal structures. c) Pressure dependence of the two shortest Te–Te bond lengths in the interlayer space.

We performed in situ high‐pressure synchrotron powder X‐ray diffraction (PXRD) measurements on NbIrTe_4_ up to 46.0 GPa. The samples used in the XRD measurements were powder ground from the single crystal samples of the same batch. The structural transition occurs at 2.4 GPa, evidenced by the appearance of new peaks in the diffraction patterns as shown in **Figure** **2**a. This transition is coincident with the resistance drop in Figure 1. Our result is quite different from the recent report results,^[^ ^22^, ^23^ ^]^ which could be rooted in sample quality and different stoichiometry. A detailed analysis of this issue is given in Table S1, Supporting Information. By performing crystal structure optimization calculations and indexing the powder diffraction patterns of the new phase, it was determined that the sample transforms from the ambient T*_d_*‐phase into a monoclinic *P*2_1_/*m* (1T′‐phase). The 1 T′‐phase can be fitted to the PXRD patterns measured at 2 GPa of the second run as shown in Figure S1, Supporting Information. The PXRD data also indicate that further increasing pressure stabilizes the monoclinic 1 T′‐phase up to 46.0 GPa, the highest pressure applied. The crystal structures of T*_d_* and 1 T′ are sketched in Figure 2b. In T*_d_* phase, the layers stack directly, resulting in a high‐symmetry orthorhombic structure. While the 1 T′ phase is a monoclinic lattice, which can be interpreted as a distortion of the 1T phase (octahedral coordination of the metal atom) through the formation of in‐plane metal‐metal atomic bonds, forming a pseudohexagonal layer with zigzag metal chains. The bulk modulus of the sample is obtained by fitting the pressure‐volume data to the third‐order Birch–Murnaghan equation of state (see Figure S3, Supporting Information). Critically, the interlayer space hedged by Te atoms shrinks continuously under pressure from 2.4 to 30.0 GPa, where the distance of nearest interlayer Te atoms decreases from 3.30 to 2.95 Å labeled as Te–Te_1 in Figure 2b,c. The second nearest distance decreases from 3.44 to 3.07 Å, labeled as Te–Te_2.

As reported, the ambient NbIrTe_4_ hosts a similar character of large magneto‐resistance (LMR) with WTe_2_ in which the nearly perfectly balanced electron–hole populations are responsible for the LMR effect,^[^ ^24^ ^]^ as shown in Figure S1, Supporting Information. However, such perfectly balanced electron–hole populations cannot coexist with superconductivity, as superconductivity was starting to be observed once LMR was totally suppressed by pressure^[^ ^13^ ^]^ and it is proved that Fermi surfaces will undergo reconstruction under pressure with the ambient two pairs of electrons and hole Fermi surfaces transforming to a single growing pair of Fermi surfaces in WTe_2_.^[^ ^25^ ^]^To further reveal how the LMR state evolves into the superconducting state in NbIrTe_4_, we systematically investigated the magnetic dependence of electrical resistance with field perpendicular to electric current of *ab*‐plane at different pressures as shown in Figure S4a–c, Supporting Information. The results indicated that the MR of NbIrTe_4_ is suppressed by the application of pressure and reaches a minimum at 2.1 GPa. At pressures higher than 2.1 GPa, MR turns to increase slightly, accompanied with emergence of superconducting state. To understand the suppression of LMR by pressurization, we conduct the in situ high‐pressure Hall coefficient (*R*_H_) measurements at low temperature (see Figure S4d,e, Supporting Information). Upon increasing pressure, the *R*_H_ undergoes a sign change from negative to positive at 4.1 GPa (see Figure S4f, Supporting Information). The temperature dependence of the Hall resistivity in ref. [26] indicated that electrons and holes coexisted for NbIrTe_4_ at ambient pressure, and electrons is notably larger than holes at low temperatures. In this work, the Hall coefficient obtained, by only considering the variation of the dominant type of carrier with pressure by single‐band model fitting. We observed evidence of superconductivity (non‐zero resistance) at 2–3 GPa, and the dominant type of carrier changes from electron to hole at 4.1 GPa. The reason is that the hole carriers are constantly increasing during the pressurization process and are in a competitive state with electron carriers at 2–3 GPa, resulting in the curve slope of hall resistance changing with magnetic field approaching zero value (as shown in Figure S4d, Supporting Information). However, at 2–3 GPa, as hole carriers are generally less than electron carriers, the hall coefficient is still negative. This could be also the reason that superconductivity does not reach zero resistance at 2–3 GPa.

The structure and superconductivity of NbIrTe_4_ under pressure was calculated by ab initio calculations combined with high‐pressure synchrotron X‐ray diffraction. Using the optimized new phase structure to calculate the electronic structure, it is difficult to see the clear surface state (see Figure S5, Supporting Information). The emergence of superconductivity in NbIrTe_4_ under pressure can be attributed to the phase transition from T*_d_*‐phase to 1 T′‐phase at around 2 GPa which introduces an inversion center and eliminates the topological Weyl fermions in the T*_d_* structure.

**Figure 3:**
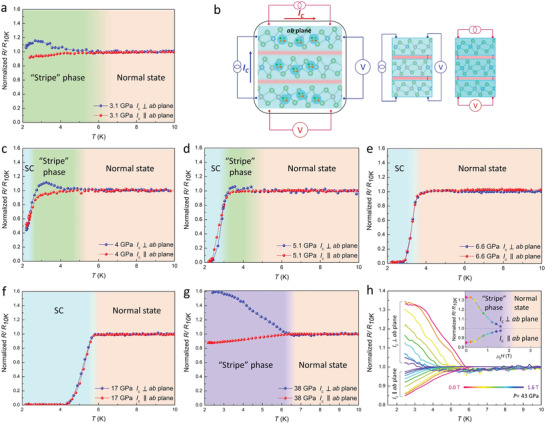
“Stripe” phase in layered NbIrTe_4_ with varying pressures. a,c–g) The NRmeasured at 3.1, 4.0, 5.1, 6.6, 17.0, and 38.0 GPa with electric current in *ab*‐plane and perpendicular to the *ab*‐plane under zero field. The blue, green (purple), and orange region indicate superconducting (SC), “stripe” phase, and normal state, respectively. b) Illustration of the measurement geometry for the case of current along the direction parallel or perpendicular to the stripes. These stripes may be composed of Cooper pairs, which are shown as blue bubbles. h) Magnetic field dependence of the resistance measured along both current directions from ≈2.5 to 10 K, from 0.0 to 1.6 T. The inset shows NR of both current directions under non‐zero field (perpendicular to *ab*‐plane) at ≈2.5 K. A “stripe” phase is revealed below the critical field (purple region).

To characterize the configuration and evolution of superconductivity in layered NbIrTe_4_ under pressure, we simultaneously measured the transporting in *ab*‐plane and perpendicular to the *ab*‐plane for the second run at high pressures (see **Figure** **3**, the other details are shown in Figure S6, Supporting Information). The measurements were carried out in a van der Pauw geometry, as shown in Figure 3b. At 3.1 GPa, we observed the emergence of a distinct state near the superconducting phase persisting over a broad temperature range from ≈6.0 K down to ≈2.0 K, revealed by an anisotropy in normalized resistance (NR) resistances of two directions and shown with green background in Figure 3a. As the temperature is just lowered than 6.0 K, the resistance increases by almost 20% for current perpendicular to the *ab*‐plane, while it decreases by over 10% for current flowing at the *ab*‐plane. The anisotropic behavior here is similar to the anisotropic superconductivity observed in a Bi_2_Sr_2_CaCu_2_O_8  +_ *_*δ*_* bulk superconductor,^[^ ^27^ ^]^ the KTaO_3_(111) interfaces,^[^ ^28^ ^]^ and the 2D granular superconductors.^[^ ^29^ ^]^ The bonding between the layers is mainly van der Waals forces, allowing NbIrTe_4_ to behave as a stack of intrinsic Josephson junctions. Unfortunately, the anisotropic effect in *ab*‐plane was not performed as the orientation cannot be determined in our current experiments. For the phase bridging the normal state and the superconductivity, its anisotropy between in‐plane and out‐of‐plane was gradually suppressed as an increase of pressure, and vanished with pressure higher than 5 GPa, where a zero resistance was reached. Again the anisotropic state was emerged when pressure higher than 33.0 GPa and persistent up to 50.0 GPa.

Figure 3h shows the resistance measured with current in *ab*‐plane and vertical to *ab*‐plane in a sample with magnetic field perpendicular to *ab*‐plane and temperature below 10 K at 43.0 GPa. With the magnetic field increasing, there is a collective increase of the resistances of *ab*‐plane below ≈6 K, exhibiting positive magnetoresistance character, while on the contrary, a collective decrease of the resistances perpendicular to *ab*‐plane exhibiting negative character. By further increasing the magnetic field, the NR of both directions tends to value “1,” as the “stripe”‐like phase is driven into the normal state (as shown in inset of Figure 3h), where the resistance anisotropy also disappears, indicating the destroying of quantum ordering in the “stripe”‐like phase.

Here we propose a “stripe”‐like phase describing anisotropic transport in the vicinity of the superconductivity due to pressure‐tuned interlayer interaction. As the intrinsic zero resistance and Meissner effect of superconducting, it is important to study the property near the boundary of phase transition. The “stripe”‐like phase is actually unbonded vortex and anti‐vortex of Cooper pairs, which could directly give the anisotropy of superconductivity in the 2D region of van der Waals layered NbIrTe_4_ and the 3D region of NbIrTe_4_ with pressure higher than 33 GPa. Important physical parameters (like upper critical fields, in‐plane and out of plane coherent lengths), the emerging and disappearing of “stripe” phase by applying pressure, and its relationship with the high temperature metal can provide useful information of the nature of superconductivity. As previous reported in some layered structure materials of topological system, such as Bi_2_Te_3_,^[^ ^30^ ^]^ Sb_2_Te_3_,^[^ ^31^ ^]^ and Bi_4_I_4_,^[^ ^32^ ^]^ there is a sign of similar anomaly before getting into the superconducting phase, which was attributed into the pressure gradient as the solid NaCl used as pressure medium. By the directional dependent resistance measurement, we proposed that such an anisotropy would naturally occur if the superconducting regions organized themselves into “stripes,” as long as the interlayer van der Waals is not strong enough to induce a 3D superconducting, as shown in Figure 3b. For this configuration, when the current is perpendicular to *ab*‐plane, the “stripes” and their vicinity are equivalent to being connected in series in the circuit, which responses to electrical conductivity of interlayers, and the current in *ab*‐plane results in parallel connected circuit, which represents electrical conductivity of intralayers. Anisotropy gradually degenerates at higher pressures (see Figure 3c,d) and the resistance in both current directions drop rapidly to zero (see Figure 3d–f) at pressure of ≈5 GPa, indicating the striped superconducting became 3D connected in single layer block or between layers next to each other by proximity effect. Unfortunately, as we did not measure the anisotropic effect of individual layer to check the stripe pattern of single layer, so the results are mainly on the interaction between different layers. At pressures above 6.6 GPa, the first “stripe”‐like phase completely disappeared. As further increase of pressure, the *T*_c_ gradually increased to a maximum of 5.8 K at pressure higher than ≈16 GPa. Generally, in polytellurides, the Te atoms can form bonds with each other, with the bond length ranging from 2.70 to 3.10 Å.^[^ ^33^ ^]^ In NbIrTe_4_, Te–Te_1, and Te–Te_2 drop below 3.10 Å above ≈17 and 30 GPa, respectively, suggesting a covalent bond formed at that pressure. Therefore, a tunnel, allowing normal electrons to be itinerant between layers, established. However, at pressures above 33 GPa, superconductivity was suppressed accompanied with the second “stripe”‐like phase in a broad temperature region (see Figure 3g). Remarkably, the normal state of the second “stripe”‐phase also shows obvious anisotropy, such as a sudden increase of resistance of *ab* plane and a sudden decrease of resistance of the vertical direction, as shown in Figure S6c, Supporting Information. We ascribe it to the combination of “dirty limit” and “weaker Josephson coupling” on the directions parallel to and perpendicular to *ab*‐plane, respectively. We noticed that the upper critical field *µ*_0_ *H*_c2_ perpendicular to *ab*‐plane increases compared with the lower pressure, as shown in Figure S6d, Supporting Information (e.g., 1.1 T of 28.0 GPa, 3.5 T of 38.0 GPa). The enhancement of *µ*_0_ *H*_c2_, which is attributed to increased electron scattering, can be understood by considering the dirty limit of superconductors.^[^ ^34^, ^35^ ^]^ Specifically, these results could be explained by the increased number of structural heterogeneity/disordering, which decreases the mean free path of electrons and thus lowers the coherence length *ξ*_*ab*_  ($\mu_{0}\;H_{c2}^{||c}$ = $\Phi_{0}\;/2\pi \xi_{ab}^{2}$) of *ab*‐plane, which in turn increases the upper critical field. With respect to the “weaker Josephson coupling” perpendicular to *ab*‐plane, it is due to the more intensive covalent bonds between the layers above 33 GPa, resulting in a sudden decrease of resistance perpendicular to *ab*‐plane (see Figure S6c, Supporting Information). Magnetic field dependence of resistance provides further evidence for superconductivity‐related “stripe”‐like phase. The decrease of resistance with magnetic field for the transport perpendicular to the “stripes” may be due to the suppression of the superconducting gap, as Cooper pairs of *ab*‐plane are disassembled into normal metallic electrons. This can be double checked while using gas pressure medium to do the transporting measurement, which was planned in our future measurement.

**Figure 4:**
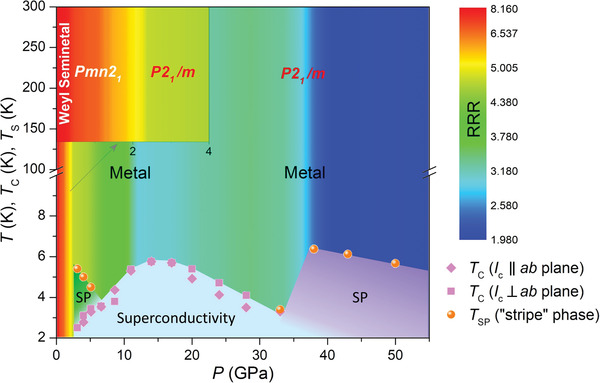
The pressure and temperature phase diagram of NbIrTe_4_. The Weyl semimetal, metal, superconductivity (blue grey region), and two different “stripe” phase (dark green and purple region) are included. The change of residual resistivity ratio RRR with pressure is represented by a gradient of color from blue to red. The inset shows the detail of phase transition from *P* mn2_1_ to *P* 2_1_/m around 2.0 GPa.

Remarkably, the evolution of residual resistivity ratio (RRR) with pressure (see Figure S7, Supporting Information) is closely related to the continuous quantum phase transitions in NbIrTe_4_. In superconducting phase, the RRR decreases steeply first accompanied with the first “stripe”‐phase emerging and enhancement of *T*_c_, and then a slightly increase of RRR accompanied with the suppression of *T*_c_. Finally, RRR decreases steeply for the second time with a second “stripe”‐phase emerging. To sum up, the pressure‐dependent RRR, two different “stripe”‐like phases and dome‐shaped superconducting phase are plotted in **Figure** **4**, where the color shading represents the value of RRR.

**Figure 5:**
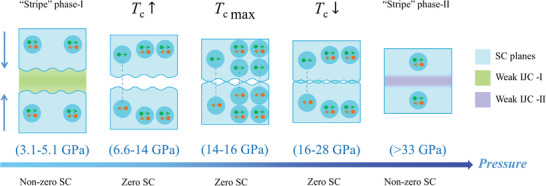
A schematic model of spatially modulated Josephson coupling under pressure. The light blue region indicates superconducting planes composed of Cooper pairs, which are shown as light blue bubbles. The green and purple regions indicate the weak interlayer Josephson coupling (IJC) regions which lead to two different “stripe”‐like phases, respectively.

The evolution of “stripes” and superconductivity with pressure can be explained by a schematic model as shown in **Figure** **5**. At pressure near 3 GPa, during the transition from normal to superconducting state, most electrons tend to look for partners at the same layer to form Cooper pairs due to the weak interlayer Josephson coupling, which is the essential cause of anisotropic character. According to the interlayer tunneling theory , the tunneling of Cooper pairs between the planes via Josephson coupling gains the kinetic energy along the *c* axis (or perpendicular to the layers), which promotes the pairing of quasiparticles and drives superconductivity.^[^ ^36^ ^]^ Generally, the Josephson coupling strength depends strongly on the interlayer spacing, as reported in the superconducting intercalated layer compound 2H‐TaS_2_,^[^ ^37^ ^]^ the expanded layers reduce Josephson coupling, resulting in a larger anisotropy ratio with coupled 2D state. On the contrary, strong Josephson coupling can lead to smaller anisotropy ratio with 3D‐like states.^[^ ^38^ ^]^ From 3.0 to ≈16.0 GPa, NbIrTe_4_ is a layered material, with van der Waals interaction, and the induced superconducting phase existing in this quasi‐2D structure. Pressure‐tuned layer spacing decreasing in NbIrTe_4_ enhanced Josephson coupling strength, which on one hand gradually weaken the “stripe”‐like phase, on the other hand promotes *T*_c_. The highest superconducting transition temperature at ≈16 GPa corresponds to a state in which the Josephson coupling strength reaches to the highest value and saturates the density of Cooper pairs. With the further reduction of the layer spacing from 16.0 to 28.0 GPa, there is coexistence of van der Waals forces and covalent bonds in NbIrTe_4_ due to the interlayer Te–Te_1 gradually forming covalent bonds between layers, which will enhance the electron scattering of the individual layers, but opens more channels for normal electrons between layers, thus gradually inhibiting the formation of Josephson coupling. Under pressure higher than 33 GPa, the covalent bonds between the layers are more strengthened due to the subsequent forming of Te–Te_2 covalent bonds, which could result in much weaker interlayer Josephson coupling, eventually a “stripe”‐like phase with significant anisotropic character. The above evolution with pressure indicates that dome‐shaped superconducting phase and anisotropic transport are all due to the spatially modulated Josephson coupling by external high pressure driven structural evolution from 2D to 3D. This schematic model may also be appropriate for explaining the pressure‐induced superconductivity with dome‐shaped *T*_c_ *‐P* phase diagram in layered structure material, such as T*_d_*‐WTe_2_^[^ ^14^ ^]^ and T*_d_*‐MoTe_2_^[^ ^16^ ^]^ in the same 1 T′ phase. The characteristics of anisotropic behavior and superconductivity associated with interlayer Josephson coupling suggests quasi 2D superconductivity in NbIrTe_4_, which needs to be further confirmed.

## 3 Conclusion

In summary, pressure‐induced dome‐shaped superconducting phase and anisotropic transport were observed in a van de Waals layered NbIrTe_4_. The sign change of *R*_H_ accompanied with suppressed MR and emergent superconductivity indicates a significant reconstruction of Fermi surface above ≈2 GPa. The characteristics of anisotropic behavior and superconductivity are associated with spatial modulation of interlayer Josephson coupling , which suggests quasi 2D superconductivity in NbIrTe_4_. It is strongly recommended to measure the exfoliated single layered NbIrTe_4_ to further confirm this proposal.

## 4 Experimental Section

In this study, all the used elements were stored and acquired in argon‐filled glovebox with moisture and oxygen levels less than 0.1 ppm, and all manipulations were carried out in the same glovebox. NbIrTe_4_ single crystals were synthesized by solid‐state reaction with the help of Te flux. The elements of Nb powder (99.99%), Ir powder (99.999%), and Te lump (99.999%) with an atomic ratio of Nb/Ir/Te = 1:1:10, purchased from Sigma Aldrich (Singapore), were loaded in a quartz tube and then flame‐sealed under high‐vacuum of 10^−6^ torr. The quartz tube was placed in a tube furnace, slowly heated up to 1000 °C and held for 100 h, and then allowed to cool to 600 °C at a rate of 1.0°C h^−1^, followed by cooling down to room temperature. The shiny, needle‐shaped NbIrTe_4_ single crystals could be obtained from the product, which displayed the layered structure.

Pressure was generated by a diamond anvil cell that consisted of two opposing anvils sitting on the supporting plates. Diamond anvils with culet diameters of 300 µm were used for this study. The T301 stainless steel gasket (thickness of 250 µm) was pre‐indented to 40 µm and then drilled with a hole of 150 µm in diameter. The four‐probe method was applied for all high‐pressure transport measurements. The high pressure Hall coefficients were measured via the Van der Paul method. The cubic BN used as the insulating layer was pressed into this hole and further drilled a small center hole with a diameter of 100 µm to serve as sample chamber, in which NaCl fine powders (for the first run) and mineral oil (for the second run) as pressure transmitting medium. High‐pressure XRD experiments were performed at CHESS of Cornell University. A monochromatic X‐ray beam with a wavelength of 0.4863 Å was chosen for XRD measurements. Pressure was determined by the ruby fluorescence method.

First‐principles calculations based on density functional theory were carried out by the Vienna ab initio simulation package.^[^ ^39^, ^40^, ^41^ ^]^ The generalized gradient approximation of the Perdew–Burke–Ernzerhof type was adopted for the exchange‐correlation potential between electrons.^[^ ^42^ ^]^ The experimental crystal parameters were carried out for all calculations with a cutoff energy of 450 eV for the plane wave expansion and a Monkhorst‐Pack 11 × 5 × 3 *k*‐point mesh was used to sample the Brillouin zone. Spin‐orbit coupling was also considered in the calculations. Real‐space tight‐binding Hamiltonian was obtained by constructing Wannier functions for the Nb‐4d orbitals, Ir‐5d orbitals, and Te‐5p orbitals using the Wannier90 package.^[^ ^43^ ^]^ The topological properties were calculated by using Wanniertools.^[^ ^44^ ^]^

## Conflict of Interest

The authors declare no conflict of interest.

## Supporting information

Supporting Information

## Data Availability

Data available on request from the authors.
