# Supplementary material for: Discovery of Dome‐Shaped Superconducting Phase and Anisotropic Transport in a van der Waals Layered Candidate NbIrTe4 under Pressure
Source: Adv Sci (Weinh). 2021 Nov 1;8(24):2103250. doi: 10.1002/advs.202103250 (PMC8693038; doi:10.1002/advs.202103250)
Supplement: Supplementary file 1 — Supporting Information [file ADVS-8-2103250-s001.pdf]

WILEY-VCH

© 2021 Wiley-VCH GmbH

ADVANCED SCIENCE
Open Access

Supporting Information

for *Adv. Sci.*, DOI: 10.1002/advs.202103250

Discovery of Dome-shaped Superconducting Phase and Anisotropic Transport in a Van der Waals Layered Candidate NbIrTe4 under Pressure

Meiling Jin[1,2†], Peng Yu[3†], Changzeng Fan[4†], Qiang Li[5], Panlong Kong[2], Zhiwei Shen[4], Xiaomei Qin[5], Zhenhua Chi[6], Changqing Jin[7], Guangtong Liu[7], Guyue Zhong[8], Gang Xu[8], Zheng Liu[9, 10*], Jinlong Zhu[1*]*

Supporting Information

Title:

Discovery of Dome-shaped Superconducting Phase and Anisotropic Transport in a Van der Waals Layered Candidate $NbIrTe_4$ under Pressure

Meiling Jin[1,2†], Peng Yu[3†], Changzeng Fan[4†], Qiang Li[5], Panlong Kong[2], Zhiwei Shen[4], Xiaomei Qin[5], Zhenhua Chi[6], Changqing Jin[7], Guangtong Liu[7], Guyue Zhong[8], Gang Xu[8*], Zheng Liu[9, 10*], Jinlong Zhu[1*]

[1]*Department of Physics & Shenzhen Engineering Research Center for Frontier Materials Synthesis at High Pressures, Southern University of Science and Technology (SUSTech), Shenzhen 518055, China*
[2]*Center for High Pressure Science and Technology Advanced Research (HPSTAR), Beijing, 100094, China*
[3]*State Key Laboratory of Optoelectronic Materials and Technologies, School of Materials Science and Engineering, Sun Yat-sen University, Guangzhou, 510275, China*
[4]*State Key Laboratory of Metastable Materials Science and Technology, Yanshan University, Qinhuangdao, 066004, China*
[5] *Department of Physics, Shanghai Normal University, Shanghai, 200234, China*
[6] *Institute of High Pressure Physics, School of Physical Science and Technology, Ningbo University, Ningbo 315211, China*
[7]*Beijing National Laboratory for Condensed Matter Physics, Institute of Physics, Chinese Academy of Sciences; School of Physical Sciences, University of Chinese Academy of Sciences, Beijing 100190, China.*
[8]*Wuhan National High Magnetic Field Center & School of Physics, Huazhong University of Science and Technology, Wuhan, 430074, China*
[9]*School of Materials Science and Engineering, Nanyang Technological University, 50 Nanyang Avenue, 639798, Singapore*
[10]*School of Electrical and Electronic Engineering, Nanyang Technological University, Singapore 639798, Singapore.*

[†]Authors contributed equally to this work
*Corresponding authors: Gang Xu: gangxu@hust.edu.cn; Zheng Liu: Z.LIU@ntu.edu.sg; Jinlong Zhu: zhujl@sustech.edu.cn

Properties at ambient pressure. $NbIrTe_4$ is a layered TMTs material and the layer stacking results in a unit cell with orthorhombic symmetry (space group of $Pmn2_1$[20]) as shown in **Figure S1a**. Nb and Ir atoms are octahedrally coordinated by Te atoms with edge-sharing octahedra extending along *a* direction forming an individual substructure layer of a 2D material. The individual layer orders in the fashion […Nb-Ir…Ir-Nb…]. The Te–Te bonds (as indicated by the dashed line in Figure 1a) between the Te–Nb(Ir)–Te sandwich layers are weak. Therefore, nanoflakes with thicknesses down to several nanometers can be exfoliated

using a scotch tape-based mechanical method. **Figure S1c, d** shows the magnetic field-dependent and angle-dependent transport measured at ambient pressure.

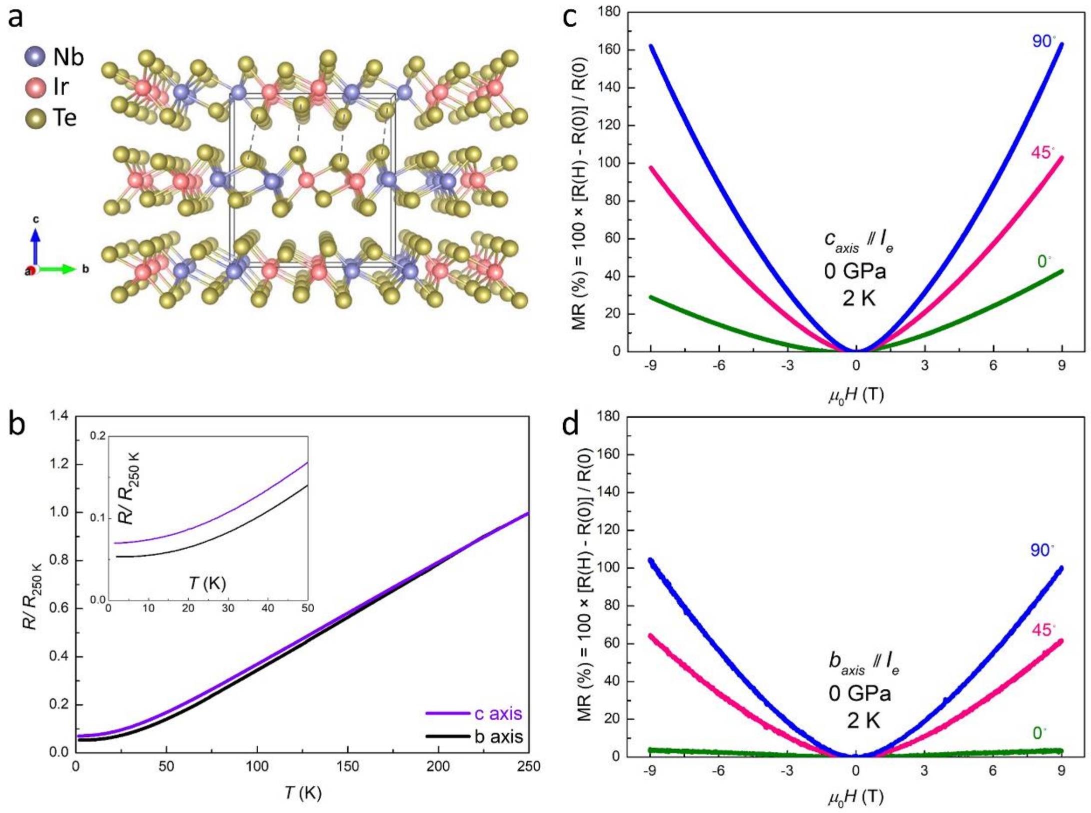

Figure S1. $NbIrTe_4$ electrical and structural properties at ambient pressure. (**a**) The atomic structure of the $NbIrTe_4$ crystal. $NbIrTe_4$ crystallizes in an orthorhombic structure, a space group of *P*mn2_1. Blue, orange and yellow spheres represent Nb, Ir and Te, respectively. (**b**) Temperature dependence of electrical resistivity of *c* axis and *b* axis at ambient pressure. The inset shows detail of data below 50 K with no hint of any superconductivity. (**c**) and (**d**) Angular dependence of the magnetoresistance with the current parallel to *c* and *b* axis at 2 K and ambient pressure. Different colors represent different angles as marked.

Comparison with existing studies. We noticed that the superconducting transition of Ref. [22] and Ref. [23] occur at much higher pressure and much lower temperature than ours. In order to more systematically analyze the characteristics of pressure-induced superconducting transition in this material system, we referred to the Weyl semimetal WTe_2 and $MoTe_2$ for comparison, which have the same crystal structure at ambient pressure, the results are shown in Table S1. We found that the experimental results on the pressure-induced superconductivity of WTe_2 reported by three groups [13-15] are not exactly the same. However, comprehensive analysis of two group's results [14, 15] indicate that the pressure-induced superconductivity in WTe_2 can be attributed to the T_d -1T' structure transition, which is consistent with our

conclusion. We consider that, for $NbIrTe_4$ or WTe_2, the inconsistency of results between different groups is related to the difference in sample quality caused by the difference in the details of synthesis. The materials in this system may be very sensitive to slight doping, which could be an interesting topic to be studied.

Table S1. Comparison of the Weyl semimetal materials with the same ambient structure.

Material	Ambient structure	Superconductivity at ambient pressure	Emergence of Superconductivity at high pressure	Structural phase transition	High pressure phase	Range of T_c	Reference
$NbIrTe_4$	T_d - ($Pmn2_1$)	No	2- 3 GPa	2- 2.4 GPa	1T′- ($P2_1/m$)	2.5- 5.6 K	This work
$NbIrTe_4$	T_d - ($Pmn2_1$)	No	27 GPa	27.5 GPa	None determined	0.5- 2.0 K	[22]
$NbIrTe_4$	T_d - ($Pmn2_1$)	No	40 GPa	None reported	None reported	2.0- 3.0 K	[23]
WTe_2	T_d - ($Pmn2_1$)	No	2.5 GPa	None reported	None reported	2.5- 7.0 K	[14]
WTe_2	T_d - ($Pmn2_1$)	No	10.5 GPa	No crystal structure transition below 20 GPa	T_d -($Pmn2_1$) below 20 GPa	2.8- 6.5 K	[13]
WTe_2	T_d - ($Pmn2_1$)	No	None reported	4– 5 GPa	1T′- ($P2_1/m$)	None reported	[15]
$MoTe_2$	T_d - ($Pmn2_1$) below 250 K; 1T′- ($P2_1/m$) above 250 K	Yes	Ambient	1.5 GPa 1T′-($P2_1/m$) above 80 K	1T′- ($P2_1/m$)	0.1- 8.2 K	[16]

Structures under high pressure. To investigate the phase transition at high pressure, we performed *in-situ* high-pressure synchrotron powder X-Ray Diffraction (PXRD) measurements on $NbIrTe_4$ up to ~30 GPa for the second run. The structural transition occurs at 2.0 GPa, evidenced by the appearance of new peaks in the diffraction patterns as shown in **Figure S2a**. The bulk modulus of the sample (of the first run) is obtained by fitting the pressure-volume data to the third-order Birch-Murnaghan equation of state (see Figure S3a). A faster nonlinear decrease in the c/a and c/b ratio (see Figure S3b) with pressure, from 2.4 to 13.2 GPa, indicates that the cell parameter c is more compressible than a and b in this pressure range as expected. Such anisotropic compressibility is attributed to the difference

between the weaker van der Waals interlayer bonding and the stronger intralayer covalent bonding.

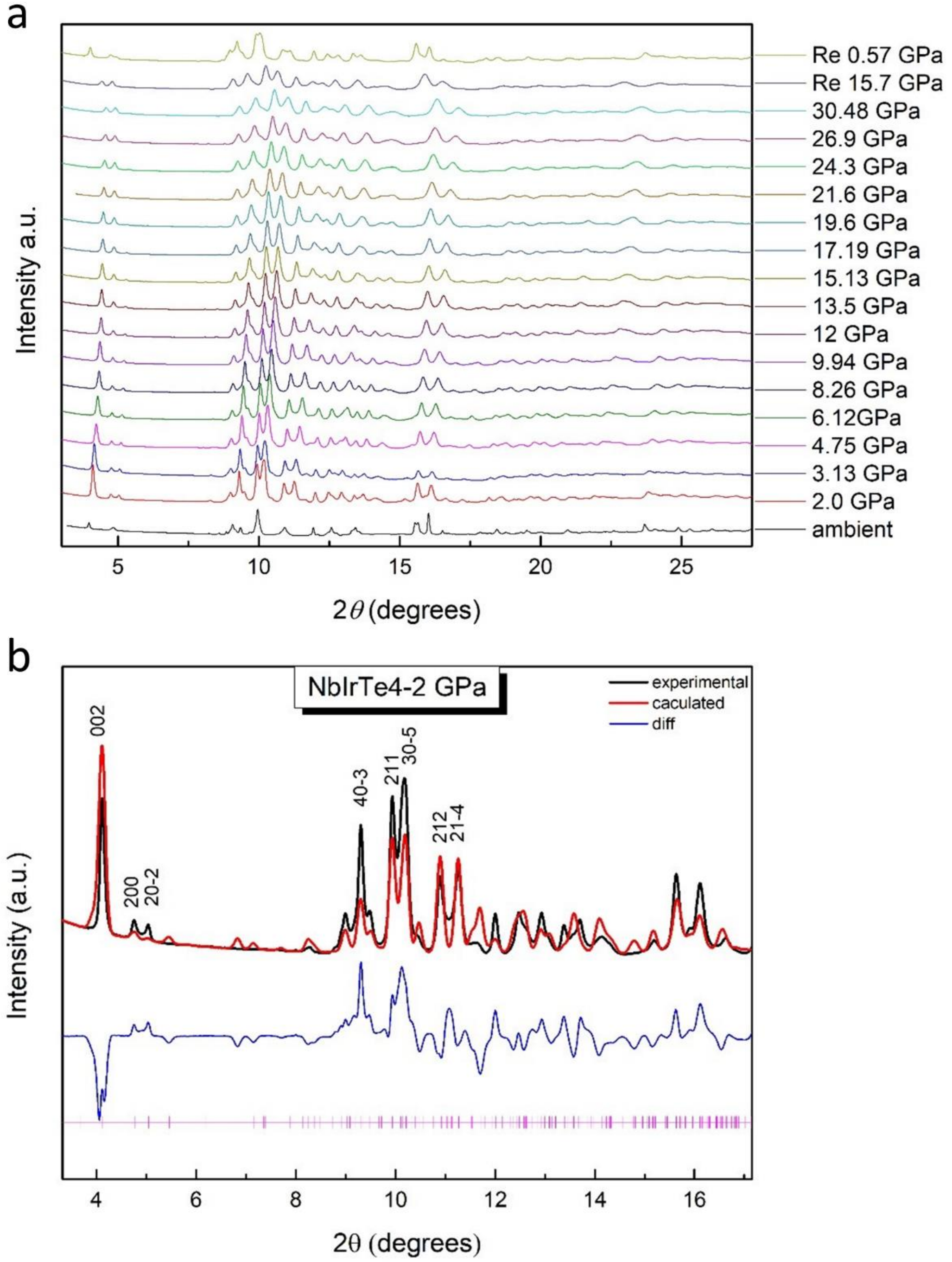

Figure S2. Synchrotron X-ray diffraction data for $NbIrTe_4$ powders of the second run. (**a**) The structural transition occurs at 2.0 GPa, evidenced by the appearance of new peaks in the diffraction patterns. (**b**) 1T′-phase 2.0 GPa/300 K. The peak positions matched well with calculated ($P2_1$/m; a=12.537 Å, b= 3.468 Å, c=14.501 Å; β= 111.299 °) Bragg peaks. The Le Bail method was used in order to fit the diffraction, as the layered $NbIrTe_4$ has preferred orientations and therefore highly textured structure, the intensity has relatively large

discrepancy.

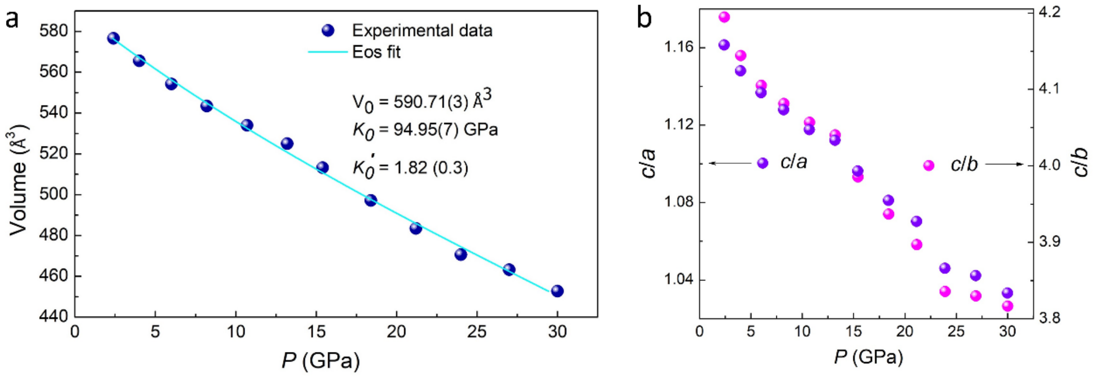

Figure S3. Synchrotron X-ray diffraction data for $NbIrTe_4$ powders (of the first run) and changes of lattice parameters at different pressures. (**a**) Volumes per formula unit as a function of pressure for $P2_1/m$ phases. The solid line is the calculated third-order Birch−Murnaghan equation of state (EoS) fit to the experimental data. (**b**) Pressure dependence of the axial ratio *c*/*a* and *c*/*b* after the structural transition.

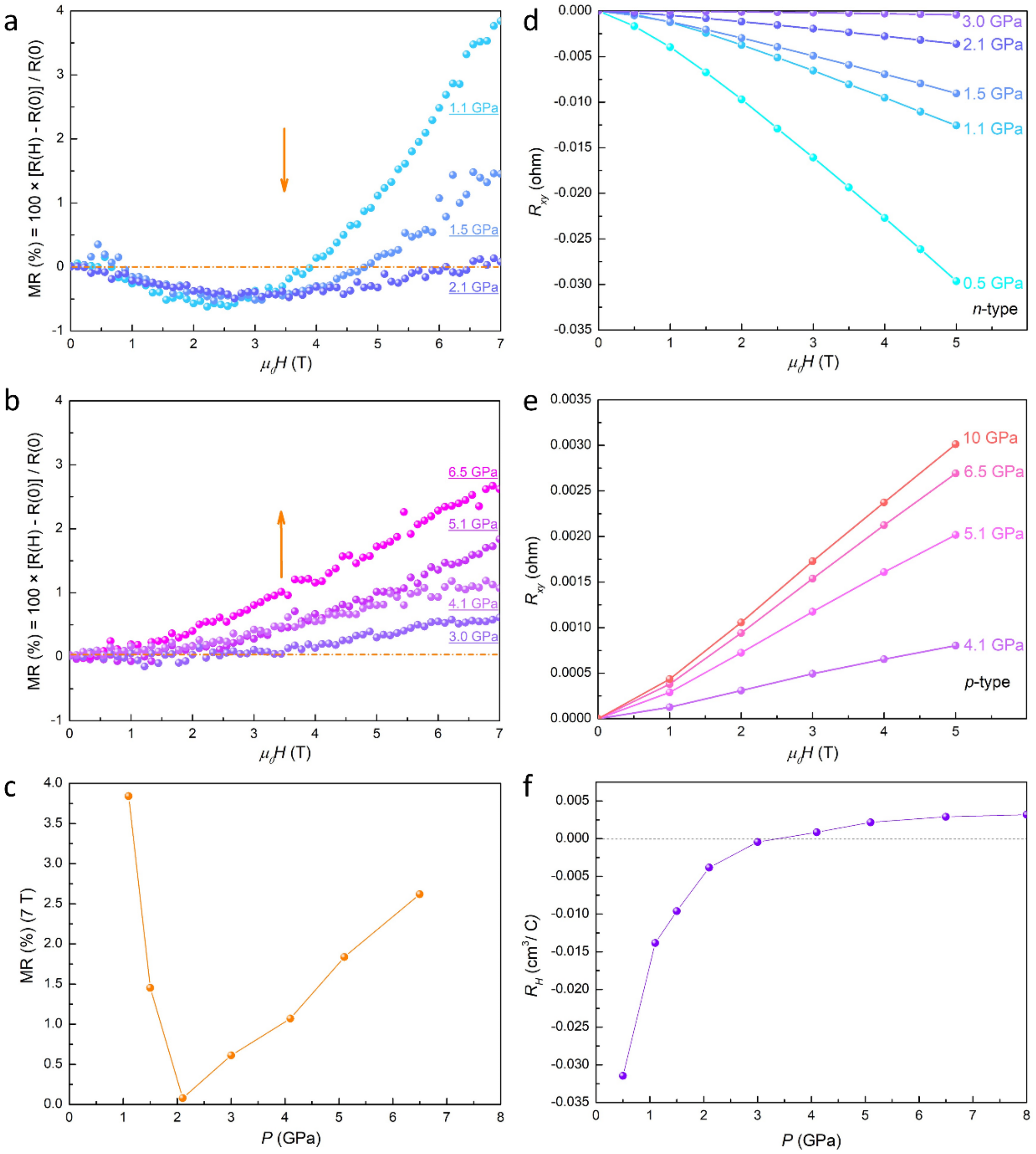

Figure S4. $NbIrTe_4$ transport measurements under external magnetic field (fixed at 10 K). (**a**) A systematic decrease of MR below 2.1 GPa. (**b**) A slightly increase of MR after the superconductivity emerges above 2.1 GPa, resulting in a minimum of MR at the critical pressure as shown in (**c**). (**d, e**) Hall resistance of $NbIrTe_4$ as a function of applied pressure, showing approximately linear behavior with a transition from a negative slope to a positive slope. (**f**) Hall coefficient R_H changes from negative to positive between 3 and 4.1 GPa.

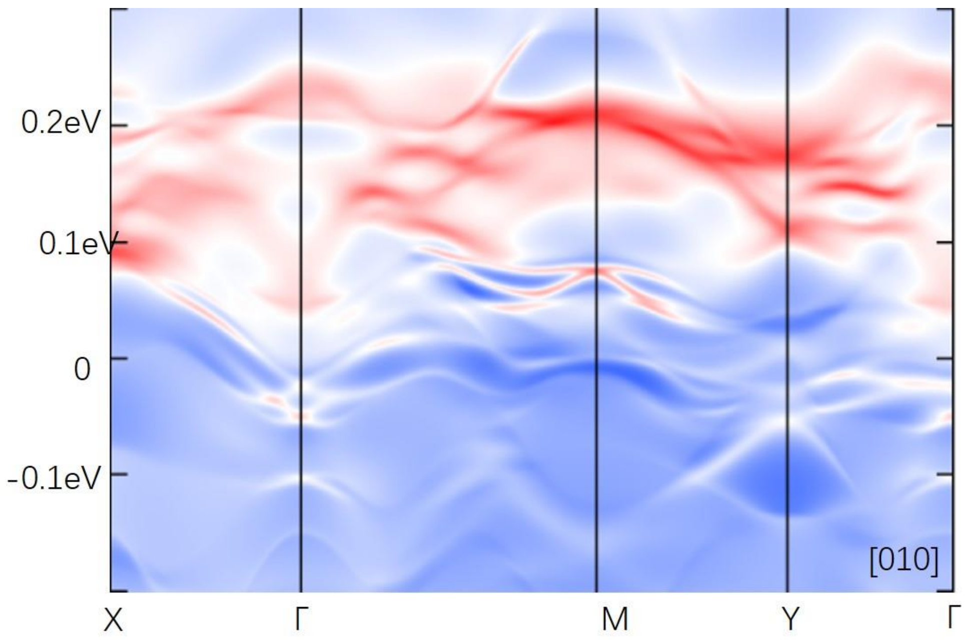

Figure S5. Calculation of electronic structure at 2.0 GPa. There is not a clear surface state, or it may have been submerged in the bulk.

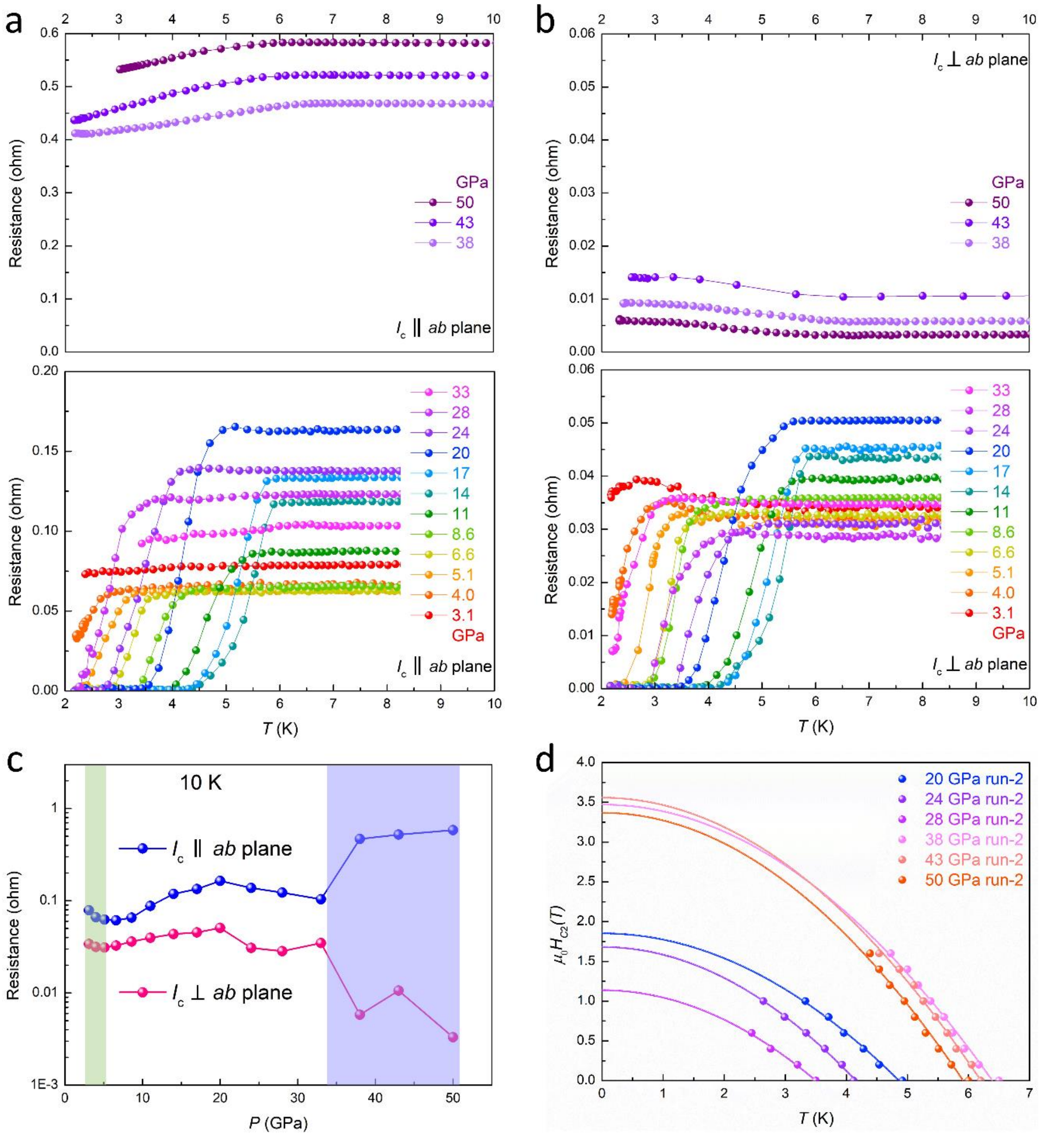

Figure S6. $NbIrTe_4$ transport measurements at high pressures of the second run. (**a**) The resistances measured at 3.1-50 GPa with electric current at *ab*-plane under zero field. (**b**) The resistances measured at 3.1-50 GPa with electric current perpendicular to the *ab*-plane under zero field. (**c**) The blue and red ball represents the resistance at 10K temperature under different pressures. The green and purple region represent two “stripe”-like phases. The resistance in both directions appears abrupt change above 33 GPa, showing strongly anisotropic character. (**d**) The fitted $\mu_0 H_{c2}$ perpendicular to *ab*-plane at different pressures of the second run. The spheres represent experimental data and the solid lines represent the WHH fits.

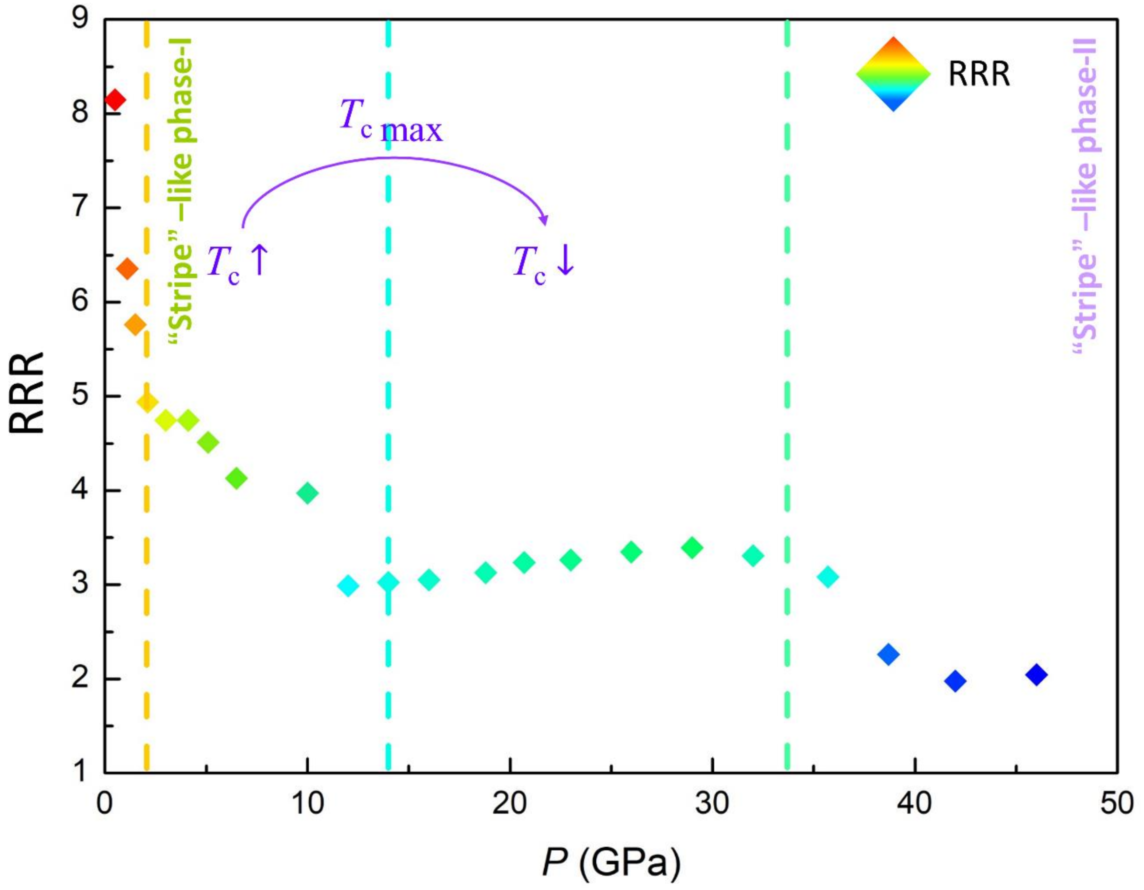

Figure S7. The change of residual resistivity ratio (RRR) with pressure. The diamond with color gradient represents the difference in RRR values.
